# Rare gallbladder adenomyomatosis presenting as atypical cholecystitis: case report

**DOI:** 10.1186/1471-230X-11-106

**Published:** 2011-10-05

**Authors:** Sheng-Hong Lin, Feng-Yee Chang, Ya-Sung Yang, Jong-Shiaw Jin, Teng-Wei Chen

**Affiliations:** 1 Department of Medicine, Tri-Service General Hospital, National Defense Medical Center, Taipei, Taiwan; 2 Division of Infectious Diseases and Tropical Medicine, Department of Medicine, Tri-Service General Hospital, National Defense Medical Center, Taipei, Taiwan; 3 Department of Pathology, Tri-Service General Hospital, National Defense Medical Center, Taipei, Taiwan; 4 Division of General Surgery, Department of Surgery, Tri-Service General Hospital, National Defense Medical Center, Taipei, Taiwan

## Abstract

**Background:**

Gallbladder adenomyomatosis is a benign condition characterized by hyperplastic change in the gallbladder wall and overgrowth of the mucosa because of an unknown cause. Patients with gallbladder adenomyomatosis usually present with abdominal pain. However, we herein describe a case of a patient with gallbladder adenomyomatosis who did not present with abdominal pain, but with only fever.

**Case presentation:**

A 34-year-old man presented to our hospital with a fever. No abdominal discomfort was declared. His physical examination showed no abnormalities. Ultrasound of the abdomen revealed thickness of the gallbladder. Acute cholecystitis was diagnosed. The fever persisted even after 1 week of antibiotic therapy. Magnetic resonance imaging of the abdomen showed gallbladder adenomyomatosis with intramural Rokitansky-Aschoff sinuses. Exploratory laparotomy with cholecystectomy was performed. The fever recovered and no residual symptoms were reported at the 3-year follow-up.

**Conclusions:**

Gallbladder adenomyomatosis can present with fever as the only symptom. Although the association between gallbladder adenomyomatosis and malignancy has yet to be elucidated, previous reports have shown a strong association between gallbladder carcinoma and a subtype of gallbladder adenomyomatosis. Surgical intervention remains the first-choice treatment for patients with gallbladder adenomyomatosis.

## Background

Gallbladder adenomyomatosis (GAM) is a degenerative disease characterized by proliferation of the mucosal epithelium and hypertrophy of the muscularis mucosae, with grossly formed mucosal invagination in the hypertrophied muscularis and radiologically apparent intramural diverticula or sinus tracts (Rokitansky-Aschoff sinuses) [1]. The most common presentation of GAM is epigastric pain; patients with GAM rarely have a fever without any other abdominal symptoms. We herein present a patient who presented with fever as the only symptom, which was determined to be caused by GAM.

## Case presentation

**Figure 1:**
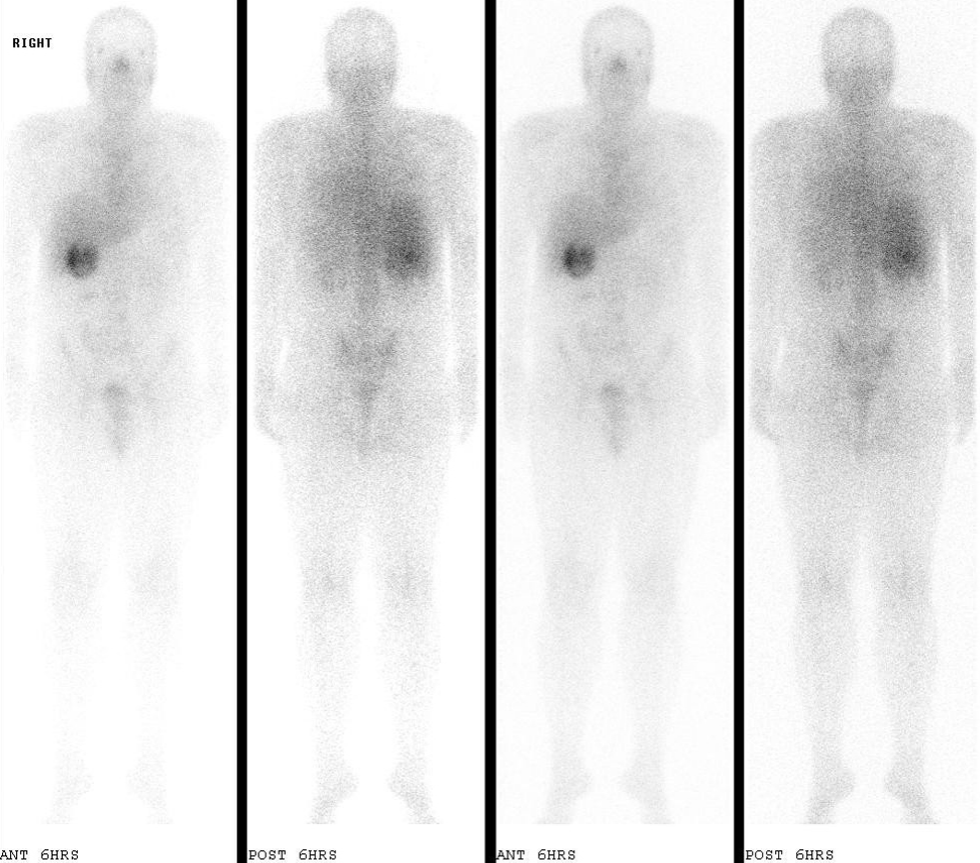
**Gallium-67 inflammation scan**. A rim-like lesion with gradually increasing intensity over the right upper quadrant of the abdomen was noticed in the gallbladder fossa.

**Figure 2:**
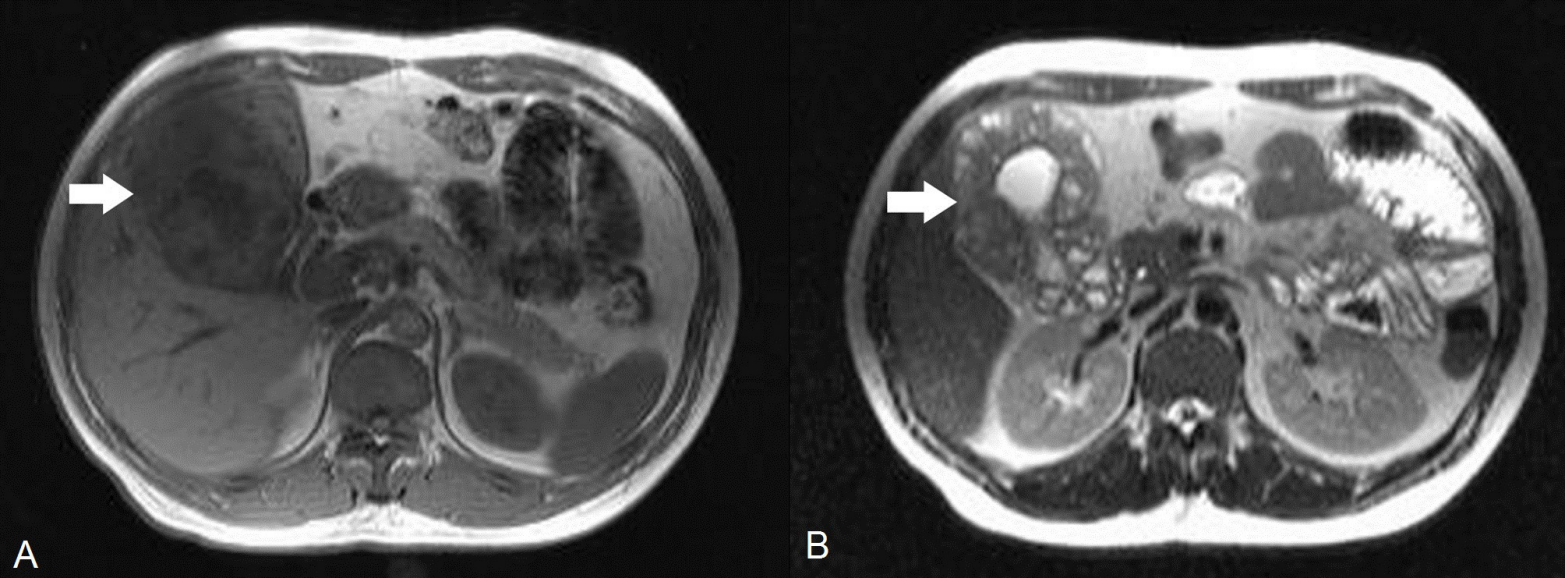
**Magnetic resonance imaging of the abdomen**. Marked thickening of the epithelial and muscular elements in the gallbladder with multiple variably sized intramural cysts (arrow) was observed in the T1- (A) and T2-weighted (B) sequences, which is consistent with adenomyomatosis of the gallbladder with intramural Rokitansky-Aschoff sinuses.

A 34-year-old man with no history of systemic disease presented with fever; he had lost 8 kg during the week before presentation. Physical examination revealed no significant findings. Laboratory investigation showed several abnormal findings, including leukocytosis (14500 cells/μL; reference range [RR], 4500-11000 cells/μL) and elevated aspartate aminotransferase (61 U/L; RR, 10-42 U/L) and alanine aminotransferase (75 U/L; RR, 10-40 U/L) levels. Ultrasonography of the abdomen revealed marked thickening of the gallbladder wall. However, serum bilirubin (0.4 mg/dL; RR, < 1.5 mg/dL), alkaline phosphatase (140 U/L; RR, 40-140 U/L), and gamma-glutamyl transpeptidase (59 U/L; RR, 8-61 U/L) levels were normal. Initially, acute atypical cholecystitis with fever as the only symptom was diagnosed, and empirical antibiotic therapy was indicated (intravenous infusion of ceftazidime [2000 mg] 3 times per day). The fever persisted even after 1 week of antibiotic therapy, and blood culture was negative. A subsequent gallium-67 scan revealed a rim-like, gallium-avid lesion in the upper right quadrant of the abdomen (Figure 1). Magnetic resonance imaging (MRI) was performed to clarify the nature of the gallbladder lesion and showed evident thickening of the epithelial and muscular elements and multiple intramural cysts of various sizes in the gallbladder wall (Figure 2). GAM with intramural Rokitansky-Aschoff sinuses was suspected; however, the possibility of a malignant gallbladder tumor could not be ruled out entirely.

**Figure 3:**
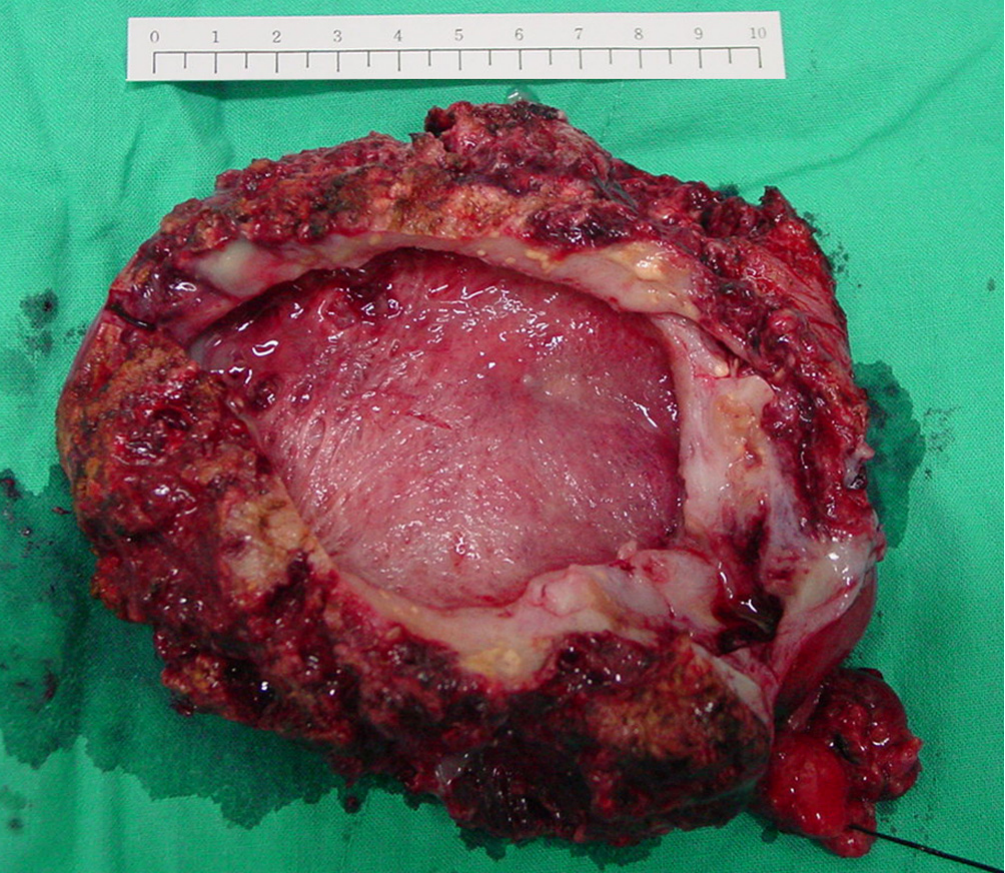
**Surgery specimen showing a huge gallbladder with multiple intramuscular cysts and abscesses**.

Exploratory laparotomy with cholecystectomy revealed an enlarged gallbladder (approximately 11 cm in diameter) with abscess formation in the gallbladder wall (Figure 3). Pathological tests revealed extensive inflammatory cell infiltration and caseous-like necrosis consisting of chronic granulomatous inflammation with acute necrotizing inflammation. Mycobacterium tuberculosis culture of the specimen revealed negative results. The fever subsided after the cholecystectomy, and the patient was discharged uneventfully 10 days after the operation. He was followed up at our outpatient department more than 3 years later; he was well and did not have a fever.

## Discussion

GAM was first described in 1960 by Jutras as a degenerative and proliferative disease of the gallbladder, and has been increasingly reported since then [1,2].

The disease was previously termed cholecystitis glandularis proliferans, cystic cholecystitis, intramural diverticulosis, adenomyoma, adenofibromyoma, hamartoma, and diverticular disease of the gallbladder. The majority of patients with GAM have reportedly been in their 50s and 60s [3]. Only a few cases of GAM in young patients have been reported, and the rate of incidence determined on the basis of cholecystectomy specimens was 2.8% to 5% [4].

The most common presentation of GAM is pain in the upper right quadrant of the abdomen, which is similar to gallstone pain with or without cholecystitis. This pain is intermittent and mostly self-limiting [5,6]. It is possible that GAM symptoms are secondary to gallstones and inflammation. Nevertheless, our patient with GAM did not present with abdominal pain. Fever was the only initial manifestation, and it persisted after 1 week of antibiotic therapy. The common symptoms of pain in the right upper quadrant of the abdomen, dyspepsia, fatty food intolerance, nausea, and vomiting that have been reported in GAM cases did not appear in our patient, either. These symptoms typically persist for a long time, but in some instances, lesions may be asymptomatic and may be incidentally discovered during radiologic investigation or autopsy [7,8]. The manifestations in our patient were similar to those in patients with acute acalculous cholecystitis (i.e., unexplained fever and leukocytosis) [2]. Therefore, our patient was diagnosed with atypical cholecystitis with fever as the only symptom.

To date, the pathogenesis of GAM has not been well understood. Neurogenic dysfunction of the gallbladder may create an increased intracystic pressure that is thought to be responsible for the formation of Rokitansky-Aschoff sinuses [3]. Furthermore, according to previous analyses, the association between GAM and gallstones ranges from 36% to 95% [9]. Chronic inflammation of the gallbladder may cause GAM. The pathological findings in our patient included chronic inflammation, but no gallstone was observed; the pathogenesis of GAM remains unclear.

Ultrasonography is an adequate, noninvasive diagnostic technique for GAM [10]. Ultrasonographic features of GAM include diffuse or segmental gallbladder wall thickening with intramural diverticula observed as anechoic spaces or as echogenic foci that may show acoustic shadows or reverberation artifacts [10]. However, ultrasongraphic diagnosis is highly operator-dependent. Ultrasound of our patient's abdomen showed gallbladder wall thickening. However, the typical findings of GAM with intramural diverticulae were not seen. Therefore, our patient was diagnosed with acute acalculous cholecystitis rather than GAM. However, because of the poor response to adequate antibiotic therapy in our patient, a further imaging study with a gallium-67 scan was indicated to survey for other infection sources. Recently, several investigators suggested that MRI is the most accurate diagnostic technique for GAM characterized by the presence of Rokitansky-Aschoff sinuses. 18F-fluorodeoxyglucose positron emission tomography for GAM may reveal hot spots, which cannot be distinguished from malignancy [11].

The key to diagnosing GAM is to identify the Rokitansky-Aschoff sinuses [11]. Some investigators have questioned the relationship between gallbladder carcinoma and GAM; it is unclear whether GAM is a pre-malignancy lesion. Ootani et al. conducted a retrospective study on 3197 consecutive cholecystectomies in which a strong association was noted between gallbladder cancer and segmental-type GAM [9]. Other types of GAM, including focal and diffuse types, did not show a strong association with gallbladder malignancy. Malignancy was excluded in our patient based on the results of the pathological examination.

Thus far, cholecystectomy has not been considered as a standard treatment for GAM. However, in symptomatic GAM patients, cholecystectomy is indicated [3,6]. The treatment for asymptomatic cases is yet to be determined. The association between gallbladder malignancy and GAM remains unclear. Some investigators continue to consider GAM to be a precursor to gallbladder malignancy, believing that stones and chronic inflammation secondary to GAM may lead to dysplasia and cancer [9]. In addition, our patient with GAM presented as a patient with acute acalculous cholecystitis. If untreated, rapid progression to gangrene and perforation may occur. Therefore, delay in surgical intervention was not recommended.

## Conclusions

This case reminded us that GAM can cause fever, which may be the only symptom in GAM, and that GAM should be included in the differential diagnoses for pyretic patients without any abdominal symptoms. Because the association between gallbladder malignancy and GAM is yet to be elucidated, cholecystectomy should be considered before conservative treatment.

## Consent

Written informed consent was obtained from the patient for publication of this case report and any accompanying images. A copy of the written consent is available for review by the Editor-in-Chief of this journal.

## Abbreviations

GAM: Gallbladder adenomyomatosis; RR: reference range; MRI: magnetic resonance imaging.

## Competing interests

There are no financial supports or other benefits from commercial sources for the work reported on in the manuscript, or other financial interests that any of the authors may have. There are no conflicts of interest to declare.

## Authors' contributions

SHL collected the data and wrote the report, and was involved in drafting the manuscript with the assistance of YSY. JSJ reviewed the histological findings. FYC and TWC made the final corrections and comments. All authors read and approved the final manuscript.

## Pre-publication history

The pre-publication history for this paper can be accessed here:

http://www.biomedcentral.com/1471-230X/11/106/prepub
